# Clothes That Kill

**Published:** 1887-09

**Authors:** 


### CLOTHES THAT KILL.

The advice to women to promote their health by out door exercises is never wanting. But no amount of fresh air exercise can save women from the evil effects of their present style of dress. It is their clothes that kill them.

Every step a woman takes her foot contends with her skirt. She lifts it on the instep, and she lifts it on the heel. The weight may be ounces or pounds, but it is taken up at every step. The heavy skirts, with flounces, overskirt, and other trimmings, hang their many pounds, flapping around the feet and legs of the wearer. The corset does not allow space to take a full breath, and the tight sleeves cause the muscles to cry for room. Dressed in this fashion, the wearer comes back from her walk for "fresh air and exercise" tired through, and through, and is the worse for it, because she has lifted and carried hundreds of pounds.

Stand at any city street corner, and watch the women as they pass. How tired they look ! How their dresses flap around them ! Contrast them with men. Men's feet lift no weight of clothes. Men's steps contend with nothing. Every muscle has its natural exercise. Out-door air and exercise are good for them.

The advice women need is for shorter, lighter and looser dresses, Mrs. Jenness Miller has not come a day too soon with her better costume. if the health of women is to be improved. Mrs. Celia Whitehead has shown "what's the matter." Before her, Mrs. Amelia Bloomer, nearly forty years ago, set the example of short loose dresses.

That style was adopted by many women, among them Mrs. Elizabeth Cady Stanton, Miss Susan Anthony, and the present writer. How light and comfortable and neat it was ! How easily we went upstairs without stepping on ourselves ! How we came downstairs without fear of being stepped on ! A walk on a rainy day or in a muddy street had no error, for there were no draggled skirts to clean. We had room to breathe, and freedom for our feet. But this healthful dress was "despised and rejected" by the great public. On one occasion, Miss Anthony, in company with me, started to go to the post-office in New York, in the Bloomer costume. But we were surrounded and wedged in by a crowd which hooted and jeered. We escaped only by a carriage sent by a friend who saw our dilemma.

It was so difficult to wear this dress, with the odium that was cast upon it, that we returned sorrowfully to the bondage of our bodies for the sake of freedom to live unmolested. That was long ago. Now women might accept the light, sensible dress which Mrs. Jenness Miller wears and commends, without fear of unpleasant comment. In it they may take fresh air and exercise, and gain in health.—*Lucy Stone.*

